# Characterization of Flexural Behavior of Hybrid Concrete-Filled Fiber-Reinforced Plastic Piles

**DOI:** 10.3390/ma17051072

**Published:** 2024-02-26

**Authors:** Sun-Hee Kim

**Affiliations:** Department of Architectural Engineering, Gachon University, Seongnam-si 13120, Republic of Korea; shkim6145@gachon.ac.kr; Tel.: +82-031-750-4718

**Keywords:** reinforcing fibers, filament winding FRP, concrete composite piles, flexural strength test, flexural stiffness

## Abstract

The reinforcing fibers in filament winding fiber-reinforced polymer (FFRP) are not arranged in the axial direction; thus, the members are vulnerable to bending and shear stresses. To address the limitations, this study evaluated FRP-concrete composite piles with reinforcing fiber arranged in circumferential directions. In particular, modular pultruded FRP (PFRP) members were fabricated with reinforcing fibers arranged in the axial and circumferential directions. The exterior of the fabricated PFRP members was reinforced with FFRP, and the flexural performance of these members was investigated through flexural strength tests. The results obtained from the flexural tests and flexural-stiffness prediction formula differed by approximately 0.72–1.36 times. A comparison between the results of the flexural test and flexural-strength prediction equation showed an error of approximately 1 to 10%.

## 1. Introduction

Industrial development and the increasing complexity of society are accompanied by more complex demands regarding the performance of structures. In recent years, the construction of structures involves understanding and satisfying the human needs for the structure rather than the approach based on conditions of the location for construction according to topography in the region and a range of environmental conditions. This trend is expected to increase rapidly with the further development of human society. However, existing construction materials should be improved to meet the conditions required in extreme environments; thus, new construction materials should be developed. Consequently, research on fiber-reinforced polymer (FRP) has actively been conducted worldwide to satisfy various conditions and requirements for structures in the construction sector. Compared to existing construction materials such as steel or concrete, FRP provides multiple physical and chemical properties advantages, such as having excellent resistance to various chemical components and not interfering with electricity and electromagnetic waves. Moreover, FRP enables the design of material with mechanical properties suitable for required functions according to the type of reinforcing fibers, matrix, or fabrication methods, to name a few. Despite such outstanding advantages of FRP as a construction material in terms of performance, its applicability remains considerably low.

To prevent the rise in carbon dioxide levels in the atmosphere due to abnormal climate events worldwide, efforts are being made to reduce emissions from human activities as much as possible. In addition, initiatives are in place to achieve carbon neutrality (Net zero) by increasing absorption and reducing net emissions to zero.

Research is being conducted on the development and use of new composite concrete that can address the carbon emissions problem associated with conventional cement concrete. The FRP used in this study offers advantages such as preventing marine pollution by resisting salt damage and corrosion. The use of FRP materials results in an enhanced durability of structures, extended useful life, and reduced carbon emissions during the construction process.

A representative member that uses FRP as a reinforcement material for concrete is the concrete-filled FRP tube (CFFT), which improves durability by reinforcing the surface with FRP. CFFT not only improves the durability of the entire member by applying FRP, which has strong chemical durability, but when used as a compression member, it confines the internal concrete and improves the load resistance performance. Thus, active research has been conducted to utilize CFFT in construction members such as piers and piles. Regarding the overseas construction industry, the concrete-filled tube (CFT) was introduced in the engineering practice of the U.K. in 1879. Since 1990, concrete-filled steel tubular columns have been applied in the ACE (architecture, construction, engineering) sector, and research on the applications of CFT has been carried out. When CFT is applied to a structure for construction, formwork is no longer required, and standardized mass production from factories is possible, achieving both constructability and cost efficiency. However, CFT faces challenges regarding joint design because the stress distribution occurring with increasing load at the interface between the concrete and steel tube cannot be determined. Additionally, if the structure where CFT is applied is located in a marine or polar environment, steel tubes may fail due to salt corrosion and freezing. In particular, CFFT is a member designed to address these problems and replace CFT by applying FRP. As CFFT uses filament winding FRP (FFRP), where the reinforcing fibers are arranged in a circumferential direction, an increase in the compressive strength may be expected from its confinement effect. However, these members can be vulnerable to bending and shear stresses. Thus, additional reinforcement materials against bending, such as rebar, are required to ensure safety in the flexural and shear behavior of the structural members, particularly for eccentric compressive loading on the FRP-concrete composite pile. Lowry [1] investigated the factors influencing the axial and lateral load responses of hollow FRP piles in soft soil by developing a numerical model to simulate the load testing of the hollow FRP piles. In particular, the findings confirmed that the number of FRP layers significantly affects the loading behavior. In contrast, the fiber orientation had a minimal effect.

Orientilize et al. [2] experimentally investigated the behavior of the spun pile-pile cap connections of damaged piles reinforced with FRP and evaluated displacement ductility. Moreover, filling the pile with rebar and concrete was effective for seismic retrofitting in the connections. Mohammed et al. [3] conducted experimental and numerical studies on the behavior of a structure repaired using prefabricated FRP composite jackets, and the results showed the effect of an increase in the tensile strength in the circumferential direction by 20% or more. Al-Jaberi et al. investigated the use of an underwater FRP system for hydraulic structures, and reported that a pre-preg (pre-impregnated materials) system was efficient for repairing dry zones. In contrast, the wet layup system was effective in repairing splash zones [4]. Almallah et al. experimentally confirmed the axial behavior of sand-coated GFRP piles in non-cohesive ground. The results showed that GFRP piles coated with silica sand improved the interfacial friction of GFRP piles in sand under axial loading and increased the pile load capacity [5]. Kim and Chung proposed a formula for calculating the bending strength of hollow concrete-filled FRP piles through analysis and experiment [6]. Hwang [7] confirmed the bearing capacity of ultra-high-strength PHC piles through load testing and confirmed that the bearing capacity was excellent. Valez and Rayhani investigated the axial and lateral load transfer of fiber-reinforced polymer (FRP) piles in soft clay and found that FRP surface topology, pile texture, and fiber weave and direction-dependent waviness pattern had a significant impact on the pile axial capacity [8]. Elhamaymy et al. experimentally assessed the durability of glass finer-reinforce polymer (GFRP)-RC piles in a marine environment against axial loads. The results showed that the axial capacities of GFRP-RC piles were enhanced by 19% compared to those of the reference baselines. These previous studies suggest that most FRP-related studies have focused on the reinforcement effect of structures for repairing and retrofitting structures [9]. Based on the background described above, this study was conducted as a follow-up study to the research of Choi et al., Lee et al., and Kang and Kim [10,11,12]. In particular, this study aims to evaluate FRP-concrete composite piles (Hybrid-CFFT, HCFFT) with a dual structure of composite materials, also used in the marine environment, with excellent flexural performance. It was manufactured to maintain the advantages of CFFT proposed in previous research [10,11,12] and to secure sufficient structural performance regarding flexibility. In this regard, HCFFT were fabricated by modular pultruded FRP (PFRP) members with reinforcing fibers arranged in the axial and circumferential directions and reinforcing the exterior of the fabricated PFRP members with filament winding FRP (FFRP). FRP members are reinforced in the longitudinal direction, so their flexural performance is weak. To compensate for this, the flexural performance was improved by wrapping the PFRP with FFRP. When FFRP is reinforced, the PFRP member resists flexure and shear. FFRP can improve axial performance by confining concrete. The mechanical properties of the material were confirmed through experiments. In addition, the flexural behavior of the HCFFT specimens was evaluated through a four-point bending test, and the flexural performance of the members was analyzed.

## 2. Mechanical Properties of HCFFT

### 2.1. Concrete Compressive Strength Test

**Figure 1:**
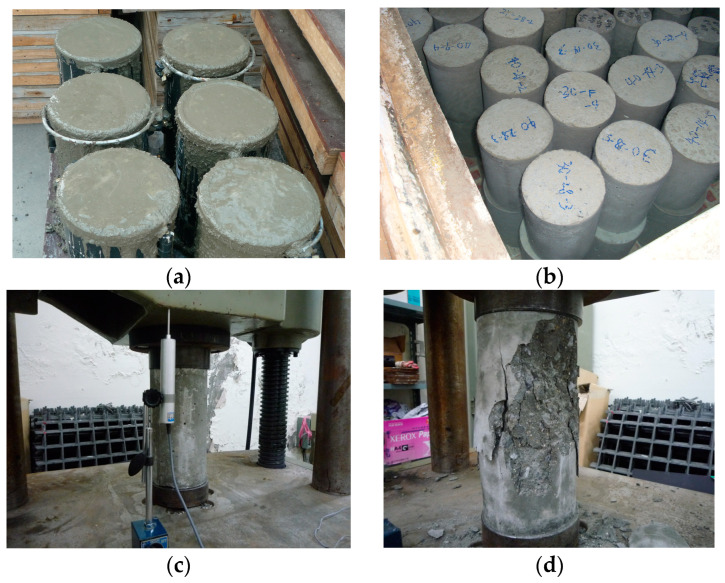
Concrete compressive strength test: (**a**) concrete specimens; (**b**) water curing; (**c**) test setup; and (**d**) failure mode.

**Figure 2:**
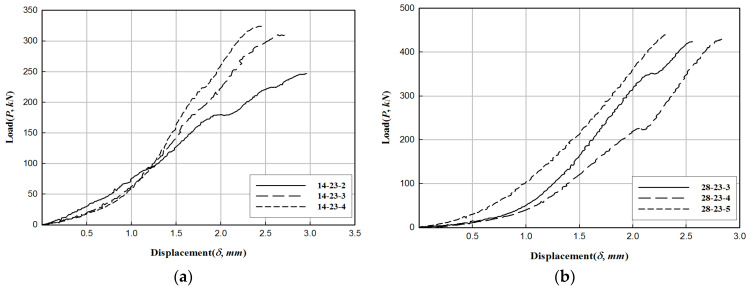
Load–displacement relationship of concrete specimens: (**a**) 14 days of water curing and (**b**) 28 days of water curing.

**Figure 3:**
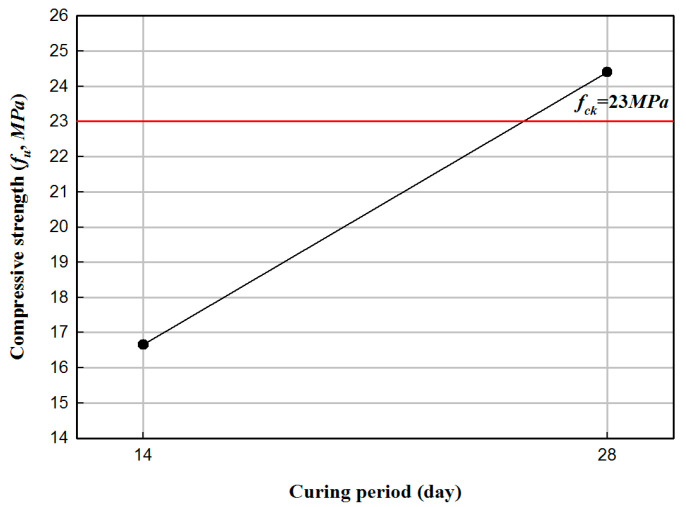
Compressive strength test with respect to the curing period.

Concrete specimens were fabricated with dimensions of 150 mm and 300 mm in diameter and height, respectively, and a compressive strength test was performed according to the Korean standard, KS F 2405 [13]. Figure 1 illustrates the key elements of the compressive strength test for concrete samples. The tests were conducted using a 1000 kN universal test machine (UTM, Japan). The load was applied at a 1 mm/min rate. Figure 1c shows the failure mode. In particular, all specimens presented cracks on the concrete surface. After 28 days, all specimens exhibited a comparable or greater compressive strength than the standard design strength. The results of the compressive strength test of concrete specimens are summarized in Table 1 and Figure 2. The change in the compressive strength of the concrete samples according to the curing period is illustrated in Figure 3. The average value of failure concrete compressive strength was calculated as the average value excluding the smallest and largest values.

### 2.2. FRP Tensile Strength Test

The tensile strength test was performed following the KS M ISO 527-4 standard [14]. The specimens used for testing were prepared by considering five of them, each from six types of cylindrical tubes with a diameter of 300 mm and layers of 4-, 6-, and 8-ply, respectively. The information on the thickness and width of the specimens is summarized in Table 2.

**Figure 4:**
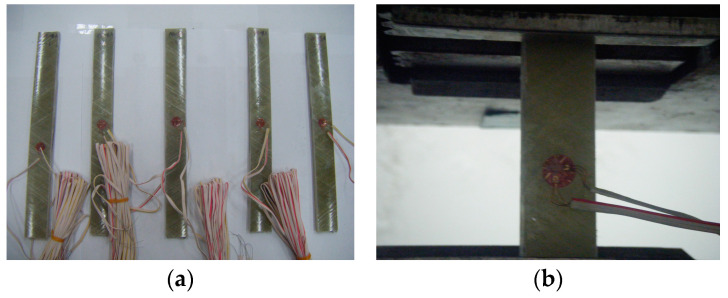
FRP tensile specimen and tensile strength test setup: (**a**) tensile specimen and (**b**) strain gauge.

For each specimen, a strain gauge was attached to the center point in the longitudinal direction and the direction orthogonal to the longitudinal direction, and the test was conducted in the UTM. The load was applied at a 3 mm/min rate in the displacement control mode. The shape of the tensile specimens and experimental setup are shown in Figure 4.

**Figure 5:**
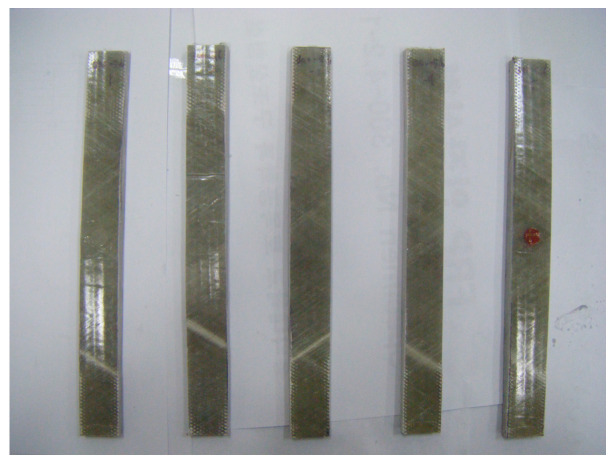
Failure mode of the tensile strength FFRP specimens.

**Figure 6:**
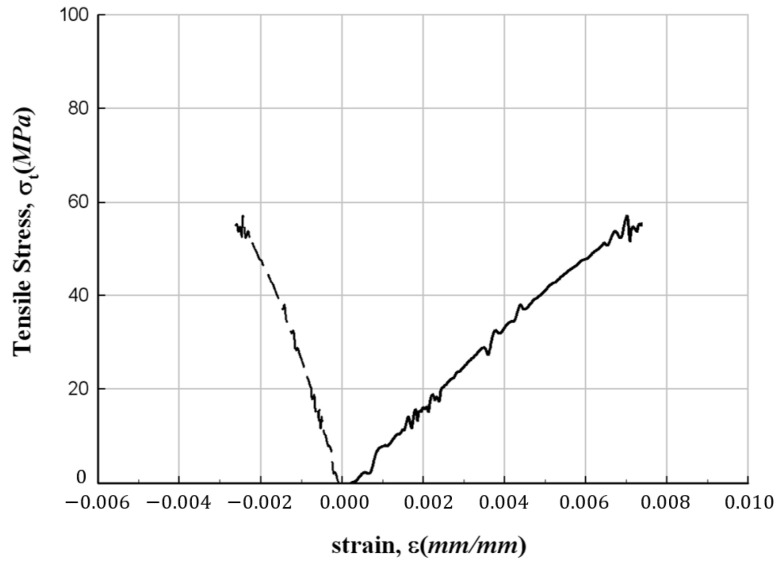
Stress–strain relationship for FFRP tensile specimen (300-28).

**Figure 7:**
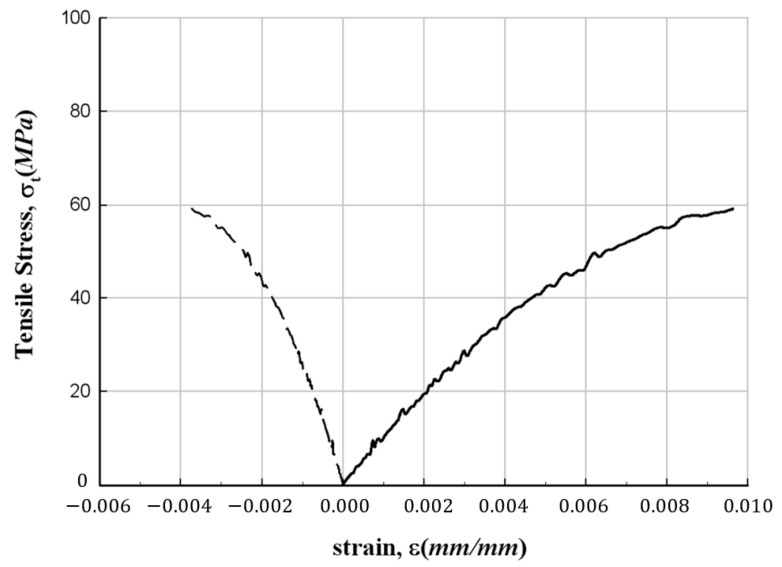
Stress–strain relationship for FFRP tensile specimen (300-42).

**Figure 8:**
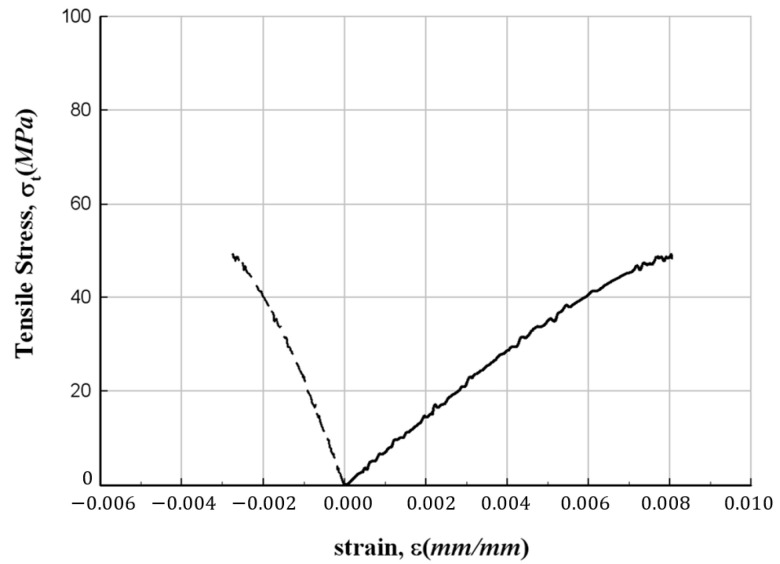
Stress–strain relationship for FFRP tensile specimen (300-56).

Figure 5 depicts the results of the tensile strength test. Notably, the failure mode of all specimens exhibits a clear fiber direction orientation. The test results are summarized in Table 3, and among the measured data, the stress–strain relationships for representative values are illustrated in Figure 6, Figure 7 and Figure 8, respectively. In these figures, the plot on the right side represents measurements from the strain gauge attached to the longitudinal direction, and the plot on the left side represents measurements from the strain gauge attached to the direction orthogonal to the longitudinal direction. The figures show that the stress increases without following a linear relationship until the point of failure of the specimens. In addition, from the figures, the longitudinal modulus of elasticity of FFRP could be determined from the slope in the strain range of 1000–3000 με by applying the method recommended in ASTM D3039/D3039M [15].

## 3. Flexural Strength Test of HCFFT Specimens

### 3.1. Cross-Sectional Design of the HCFFT Specimens

**Figure 9:**
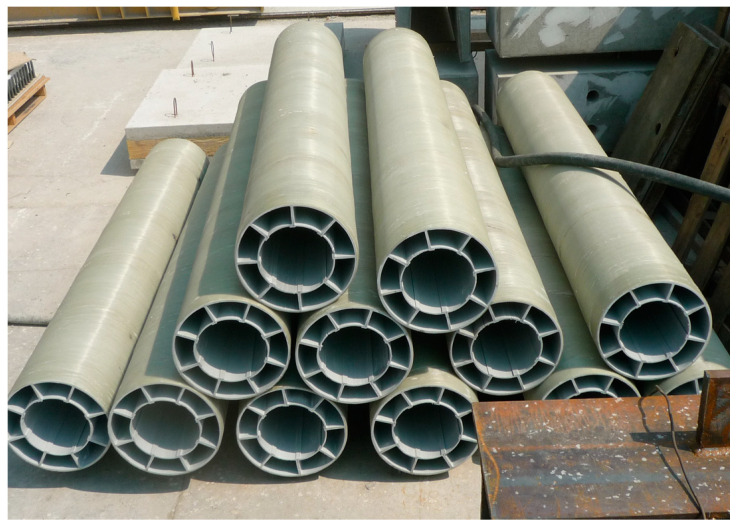
Cross-section shape.

The design of the cross-section of the HCFFT specimens was determined based on the results of compressive strength testing of CFFT obtained by Choi et al. and Kang and Kim [10,12]. The geometry of the cross-section of HCFFT is illustrated in Figure 9. A total of 45 experimental data points were obtained from the HCFFT specimens. The results indicated that the data showed a linear distribution. Thus, a linear regression analysis was performed with the data. Furthermore, similar to the case of CFFT, the relationship between the confinement and strength ratios was obtained from the slope of the fitted line. Equation (1) presents the fitted equation [8].

(1) $$\frac{f_{cc}}{f_{co}}=1.00+3.89\frac{f_{l}}{f_{co}},$$

where *f_cc_* represents the compressive strength of the laterally confined concrete column, *f_co_* is the compressive strength of the column without lateral unconfinement, and *f_l_* is the force of lateral confinement.

**Figure 10:**
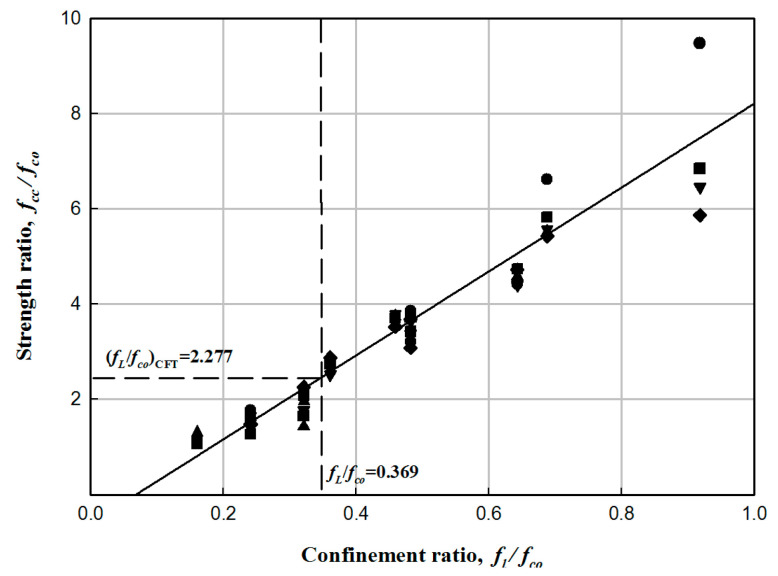
Confinement ratio of the flexural strength test specimen.

The cross-section of the HCFFT flexural specimen was set as a circle with a diameter of 300 mm. Moreover, the strength ratio-confinement ratio relationship for the CFFT specimens is shown in Figure 10. The confinement ratio of the CFFT compressive test specimen with the same strength ratio as that of the CFT cross-section presented in the previous study was determined.

The concrete standard design strength of the HCFFT flexural specimen and the thickness of FFRP were determined at similar values as that of the compressive strength ($P_{CFT}$ = 4830 kN) of CFT presented in the previous study, considering the compressive strength of CFFT that can be obtained from Equation (1) and the compressive strength of PFRP.

The concrete standard design strength of CFFT, with the same compressive strength as that of CFT, and the thickness of FFRP were 23 MPa and 2.8 mm (4 ply), respectively. Regarding the FFRP, manufacturing the products with a thickness of 3 plies or lower was challenging; therefore, the variable of FFRP thickness for the specimen was set to 2.8 mm (4 ply), 4.2 mm (6 ply), and 5.6 mm (8 ply), which are the same as that of the compressive strength test specimen. The dimensions, variables, and quantity of the fabricated HCFFT flexural specimen are listed in Table 4.

### 3.2. Flexural Strength Test

**Figure 11:**
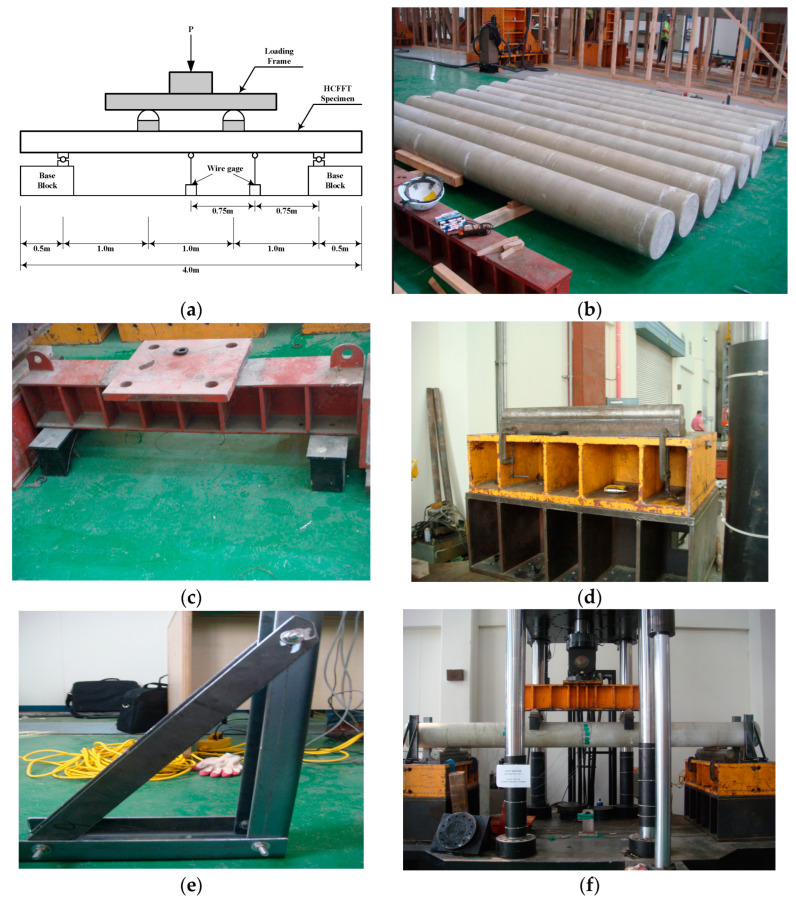
Flexural strength test setup for the HCFFT specimen: (**a**) loading method in the HCFFT flexural strength test; (**b**) HCFFT specimen; (**c**) loading frame; (**d**) supports; (**e**) fixed device; and (**f**) setup.

Figure 11a shows that the specimens in the experiments were positioned as an overhanging beam extending 500 mm both to the left and right. Moreover, the same load was applied to 1/3 and 2/3 points so that pure bending occurs in these specimens. The specimens are shown in Figure 11b, the loading frame in Figure 11c, the supports in Figure 11d, and the fixed device in Figure 11e. The fixed device in Figure 11e was installed to eliminate pile movement during the experiment. For each specimen, as shown in Figure 11f, seven and five strain gauges were attached to the center and 1/4 points, respectively, in the longitudinal direction, and wire gauges with a capacity of 1000 mm were attached to the center point and 1/4 point, one at each point, to measure the displacement by lateral loading. The load was applied using the 2000 kN UTM at a 5 mm/min rate using the displacement control mode.

## 4. Results of the Flexural Strength Test of HCFFT Specimens and Analysis

**Figure 12:**
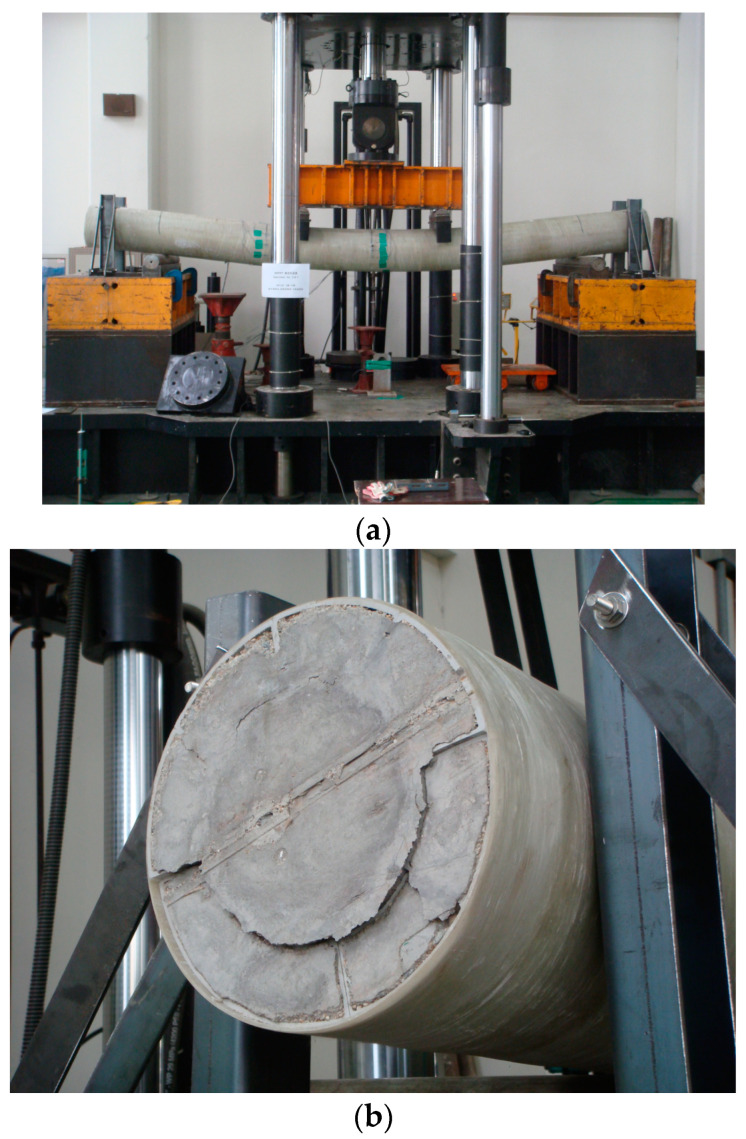
Failure mode of the HCFFT flexural specimen: (**a**) flexural failure mode of HCFFT center and (**b**) deformed shape at the end of the specimen.

**Figure 13:**
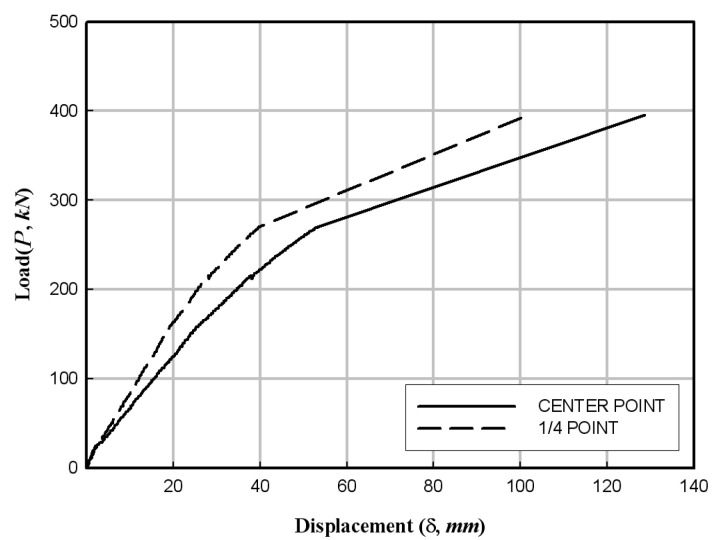
Load–displacement relationship of the HCFFT flexural specimen (2.8-2).

**Figure 14:**
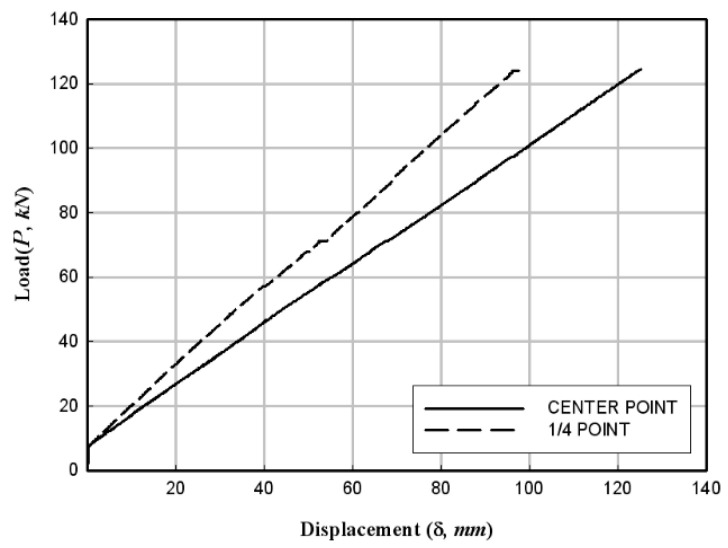
Load–displacement relationship of the HCFFT flexural specimen (4.2-2).

**Figure 15:**
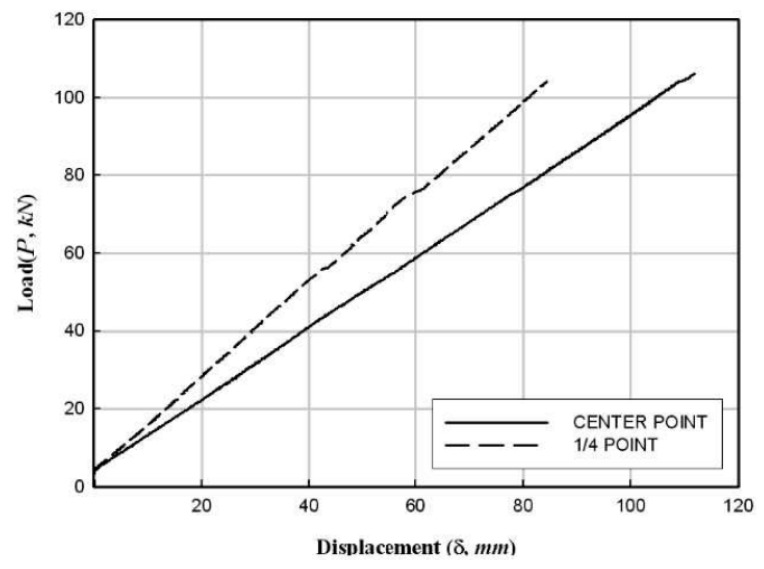
Load–displacement relationship of the HCFFT flexural specimen (5.6-2).

Figure 12a shows that the flexural failure occurred in the central section of the specimens. Moreover, the FFRP in the tension zone around the center of the specimens was delaminated in the direction of fiber orientation/arrangement. In addition, the PFRP tended to be pushed to the inner side of the member at the end sections of the specimens. Figure 12b shows the deformed shape at the end of each specimen. The experimental results are summarized in Table 5, and the load–displacement relationships obtained from the data measured from wire gauges are shown in Figure 13, Figure 14 and Figure 15.

**Figure 16:**
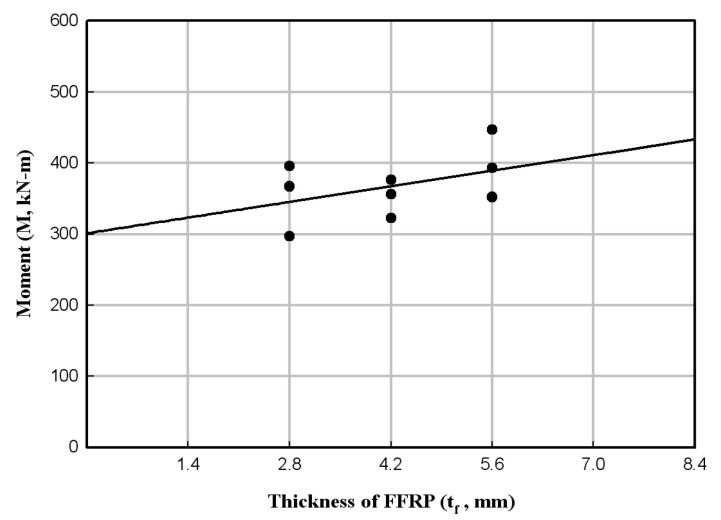
Changes in the bending moment at failure with respect to the thickness of FFRP.

According to the flexural strength test of the HCFFT specimens, the flexural strength linearly increased when regression analysis was performed between the thickness of the FFRP and flexural strength for each set of experimental results. Figure 16 shows the changes in the bending moment at failure and the trend line with respect to the thickness of the FFRP. However, as the increase in flexural strength was minimal, and the deviation of data was large, additional experiments should be performed.

The neutral axis was located from the strain gauges attached to the center point and 1/4 point of the HCFFT flexural specimen. Moreover, the flexural stiffness of the member was obtained, and the experimental results were compared with the prediction formula for flexural stiffness. Table 6 lists the results of the flexural stiffness obtained experimentally and by the prediction formula. In this study, the effective flexural stiffness applied to the concrete-filled steel pipe structure in AISC 360 [16] for application to HCFFT was proposed as shown in Equation (2).

The results indicated that the values obtained by the prediction formula and those obtained experimentally differ approximately 0.72 to 1.36 times. Therefore, the results suggest that Equation (2) needs to be reconsidered to apply it as a prediction formula for design purposes. In Equation (2), ${EI}_{HCFFT}$ represents the prediction formula for the flexural stiffness of HCFFT.

(2) $${EI}_{HCFFT}=\frac{EI}{0.72}=1.39(E_{ffrp}I_{ffrp}+E_{pfrp}I_{pfrp}+C_{H}E_{c}I_{c}),$$

where, E_ffrp_ is the elastic modulus of filament winding FRP, I_ffrp_ is the moment of inertia of the area of filament winding FRP, E_pfrp_ is the elastic modulus of pultruded FRP, I_pfrp_ is the moment of inertia of the area of pultruded FRP, C_H_ is the reduction coefficient corresponding to HCFFT, E_c_ is the elastic modulus of concrete, and I_c_ is the moment of inertia of the area of concrete.

The research results of Choi et al. [10] confirmed that the compressive strength of HCFFT is higher than that of CFFT because FFRP reinforced PFRP. The research results of Lee et al. [11] confirmed that it is economical to design an FFRP with a thickness of 4.2 mm or less, and that the increase in strength decreases when it exceeds 4.2 mm.

During the flexural testing of the HCFFT specimens, the attachment between PFRP and FFRP deteriorates. Therefore, the attachment strength of PFRP and FFRP can be determined by using the bending moment at the time of detachment between PFRP and FFRP. Table 7 lists the values of the maximum bending stress (attachment strength) generated in the cross-section when the PFRP and FFRP detached in each specimen.

The flexural strength of HCFFT can be predicted by applying the equilibrium relationship between the tensile and compressive forces within the cross-section due to the bending moment. 

As concrete is confined by FFRP, the confinement coefficient obtained in the previous study [12] was applied. As PFRP cannot consider the ribs of the cross-section owing to changes in the neutral axis, the compressive strength was considered by replacing it with a ring-shaped cross-section with the same cross-sectional properties. The flexural strength equation of HCFFT is as Equation (3).

(3) $$M_{HCFFT}=\frac{f_{pfrp}D^{2}}{8}\left[\left(2\alpha -\pi \right)\left(\frac{\alpha \sin\theta }{\theta \left(\pi -\alpha \right)}+\frac{\sin\theta }{\theta }\right)+\left(\pi -\alpha \right)\left(\frac{\alpha \sin\theta }{\theta \left(\pi -\alpha \right)}+\frac{2{sin}^{3}\theta }{3\left(\theta -\sin\theta \cos\theta \right)}\right)\right]$$

where D is the diameter of the cross-section, and θ is the angle between the straight line perpendicular to the center of the cross-section and the radius meeting the neutral axis. α is a parameter for θ. α is shown in Equation (4).

(4) $$\alpha =\theta -\frac{1}{2}\sin\theta$$

Additionally, in Equation (3), θ was calculated numerically using the equilibrium relationship between tensile and compressive force. The results of the flexural test and flexural-strength equation are compared in Table 8, and an error of up to 10% is observed. 

## 5. Conclusions

In this study, the structural behavior of HCFFT was examined with a four-point bending test to evaluate the flexural strength of HCFFT. Regarding the mechanical properties of HCFFT, the compressive strength of concrete was 23 MPa. This value corresponds to 106.10% of the standard design strength. When examining the tensile strength with respect to the thickness of FRP, the average tensile strength of FRP was 63.23 MPa when the thickness was 2.8 mm, which was higher than the tensile strength for the thickness of 6 ply and 8 ply. Moreover, the reliability of HCFFT was verified by characterizing the mechanical properties by conducting flexural strength tests, and a prediction formula for flexural stiffness was proposed. The result obtained from the proposed prediction formula for flexural stiffness showed a difference of 11% compared to the flexural stiffness obtained experimentally. In addition, the attachment strength between PFRP and FFRP decreased when the thickness of FFRP was 6 ply (4.2 mm) and increased when the thickness of FFRP was 8 ply (5.6 mm). When the results of the HCFFT flexural test and flexural-strength prediction equation were compared, errors of 1 to 10% were observed. Given the increasing demands for improving the performance of structures due to industrial development and the increasing complexity of society, the developed HCFFT and prediction equation can contribute to advances in the design of structures.

## Figures and Tables

**Table 1:** Result of concrete compressive strength test (*f_ck_* = 23 MPa).

Curing Period (Day)	Specimen No.	Failure Concrete Compressive Strength (MPa)	Average Failure Concrete Compressive Strength (MPa)	$f_{max.ave}/f_{ck}$ (%)
14	23-14-1	13.76	16.65	72.40
	23-14-2	13.97		
	23-14-3	17.52		
	23-14-4	18.46		
	23-14-5	24.81		
28	23-28-1	31.29	24.40	106.10
	23-28-2	17.17		
	23-28-3	23.98		
	23-28-4	24.29		
	23-28-5	24.92		

**Table 2:** Dimensions of FRP specimens for tensile strength test.

Specimen No.	Width (mm)	Thickness (mm)	Area (mm^2^)
300-28-1	25.52	3.13	79.88
300-28-2	26.35	2.50	65.88
300-28-3	26.07	3.29	85.75
300-28-4	26.07	3.53	92.03
300-28-5	24.42	3.52	85.96
300-42-1	25.41	6.51	165.42
300-42-2	25.89	6.18	160.00
300-42-3	25.31	6.76	171.06
300-42-4	26.43	6.16	162.78
300-42-5	24.75	5.88	145.53
300-56-1	26.07	8.71	227.03
300-56-2	25.52	8.19	209.01
300-56-3	26.78	8.56	229.24
300-56-4	25.00	8.84	220.96
300-56-5	27.70	8.82	244.27

**Table 3:** Result of the tensile strength tests.

Specimen No.	$f_{max}$ (MPa)	$E_{11}$ (GPa)	$\nu_{12}$
300-28-1	62.71	9.46	0.34
300-28-2	73.42	-	-
300-28-3	62.71	11.37	0.36
300-28-4	60.14	10.23	0.36
300-28-5	57.16	8.45	0.32
Average	63.23	9.88	0.35
300-42-1	52.17	7.63	0.36
300-42-2	55.43	-	-
300-42-3	52.22	7.05	0.36
300-42-4	59.40	9.09	0.37
300-42-5	62.14	11.29	0.36
Average	56.27	8.77	0.36
300-56-1	50.31	9.54	0.38
300-56-2	52.57	9.87	0.41
300-56-3	50.62	10.53	0.42
300-56-4	48.18	8.23	0.41
300-56-5	49.27	7.16	0.33
Average	50.19	9.07	0.39

**Table 4:** Description of the HCFFT flexural specimens.

Diameter (*D*, mm)	300		
Length (*L*, mm)	4000		
Concrete Design Standard Strength (*f_ck_*, MPa)	23		
PFRP Thickness (*t_p_*, mm)	1.0$t_{p}$		
FFRP Thickness (*t_f_*, mm)	2.8 (4 ply)	4.2 (6 ply)	5.6 (8 ply)
Specimen	3	3	3

**Table 5:** Results of the HCFFT flexural strength test.

Specimen No.	FFRP Thickness (tf, mm)	Ultimate Load (Pu, kN)		Moment at Failure (Mu, kN·m)		Max. Displacement (δmax, mm)		1/4 Point Maximum Displacement (δ_1_/4, mm)	
		Specimen	Ave.	Specimen	Ave.	Specimen	Ave.	Specimen	Ave.
2.8-1	2.8	367.0	353.1	367.0	353.1	149.9	129.2	112.9	98.2
2.8-2		395.3		395.3		130.1		101.6	
2.8-3		297.1		297.1		107.5		80.2	
4.2-1	4.2	376.2	351.6	376.2	351.6	132.5	135.4	96.1	102.3
4.2-2		356.0		356.0		148.5		112.1	
4.2-3		322.5		322.5		125.2		98.6	
5.6-1	5.6	392.8	397.2	392.8	397.2	114.9	114.9	84.8	84.6
5.6-2		351.9		351.9		111.9		84.4	
5.6-3		446.8		446.8		117.8		84.7	

**Table 6:** Flexural stiffness of the HCFFT specimens.

Specimen No.	FFRP Thickness $(t_{f}$, mm)	Flexural Stiffness				
		Experimental Result (kN m)		Predicted Values (kN m)	Predicted Values/Experimental Result (%)	
		Specimen	Average		Specimen	Average
2.8-1	2.8	10,515.21	9192.58	8011.92	0.76	0.87
2.8-2		11,155.50			0.72	
2.8-3		5907.04			1.36	
4.2-1	4.2	9287.29	9308.34	8250.81	0.89	0.89
4.2-2		8134.95			1.01	
4.2-3		10,502.78			0.79	
5.6-1	5.6	11,422.55	11,302.59	8488.55	0.74	0.75
5.6-2		11,140.29			0.76	
5.6-3		11,344.93			0.75	

**Table 7:** Attachment strength between PFRP and FFRP.

Specimen No.	FFRP Thickness $(t_{f}$, mm)	Attachment Strength (MPa)	
		Specimens	Ave.
2.8-1	2.8	134.66	129.56
2.8-2		145.02	
2.8-3		109.00	
4.2-1	4.2	136.14	127.21
4.2-2		128.80	
4.2-3		116.70	
5.6-1	5.6	140.19	141.75
5.6-2		125.59	
5.6-3		159.46	

**Table 8:** Comparison between HCFFT flexural test and flexural-strength equation results.

Description	FFRP Thickness		
	*T_ffrp_* = 2.8 mm	*T_ffrp_* = 4.2 mm	*T_ffrp_* = 5.6 mm
Flexural test results (kN·m)	353.1	351.6	397.2
Numerical results (kN·m)	356.3	358.3	360.7
Error (%)	0.99	0.98	1.10

## Data Availability

Data are contained within the article.
